# Supplementary material for: Nonvolatile optical phase shift in ferroelectric hafnium zirconium oxide
Source: Nat Commun. 2024 May 9;15:3549. doi: 10.1038/s41467-024-47893-2 (PMC11082191; doi:10.1038/s41467-024-47893-2)
Supplement: Supplementary file 1 — Supplementary Information [file 41467_2024_47893_MOESM1_ESM.pdf]

Supplementary Information

Nonvolatile optical phase shift in ferroelectric hafnium zirconium oxide

Kazuma Taki[1], Naoki Sekine[1], Kouhei Watanabe[1], Yuto Miyatake[1], Tomohiro Akazawa[1], Hiroya Sakumoto[1], Kasidit Toprasertpong[1], Shinichi Takagi[1], and Mitsuru Takenaka[1*]

[1] Department of Electrical Engineering and Information Systems, The University of Tokyo, 7-3-1 Hongo, Bunkyo-ku, Tokyo 113-8656, Japan

*E-mail: takenaka@mosfet.t.u-tokyo.ac.jp

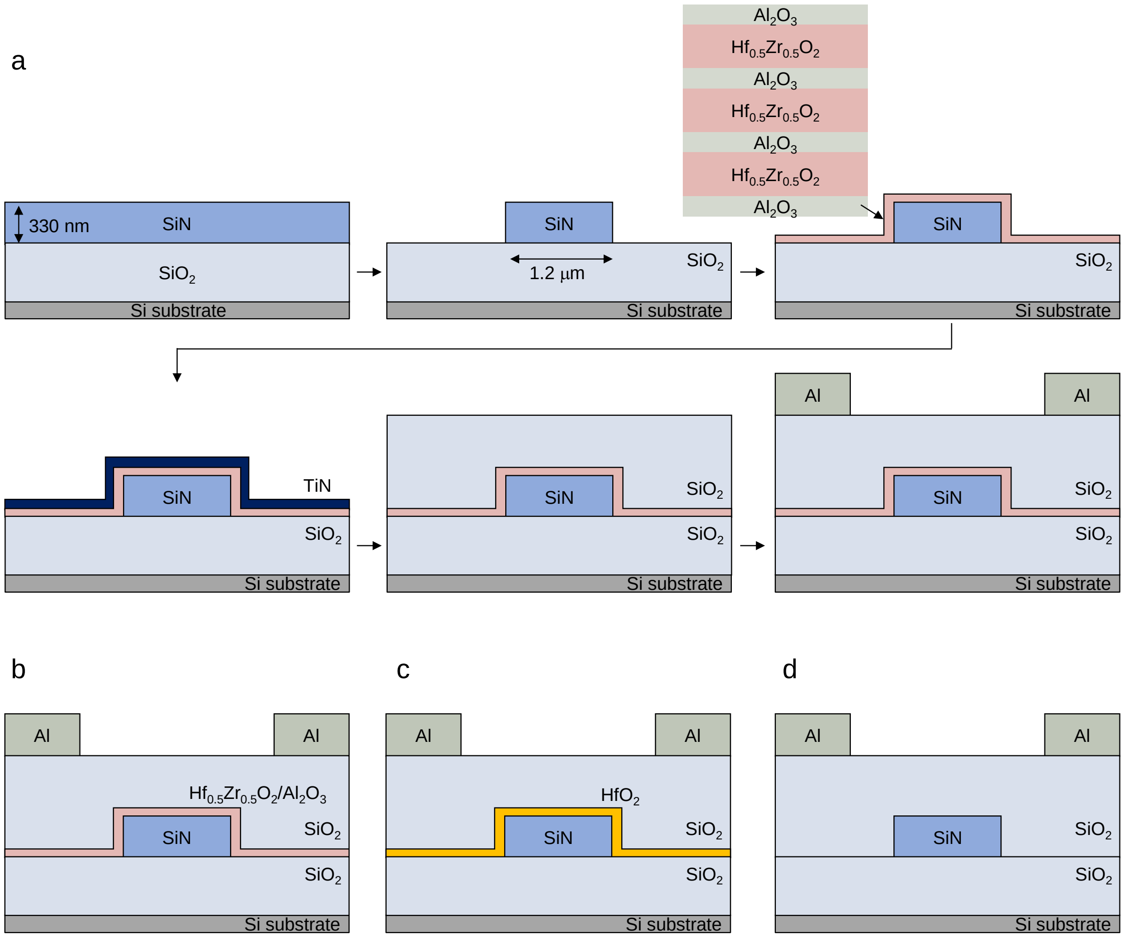

Fig. S1. **Device fabrication. a,** Fabrication procedure for the optical phase shifter with $Hf_{0.5}Zr_{0.5}O_2/Al_2O_3$ stacks deposited on the SiN waveguide. After depositing a SiN layer on a thermally oxidized Si wafer, SiN waveguides are formed. After depositing $Hf_{0.5}Zr_{0.5}O_2/Al_2O_3$ stacks by ALD, TiN is deposited followed by annealing at 400 °C for 1 min. After removing TiN, SiO_2 cladding and Al electrodes are deposited. **b, c, d,** Cross-sectional schematics of the SiN waveguide with the $Hf_{0.5}Zr_{0.5}O_2/Al_2O_3$ stacks, HfO_2, and SiO_2, respectively.

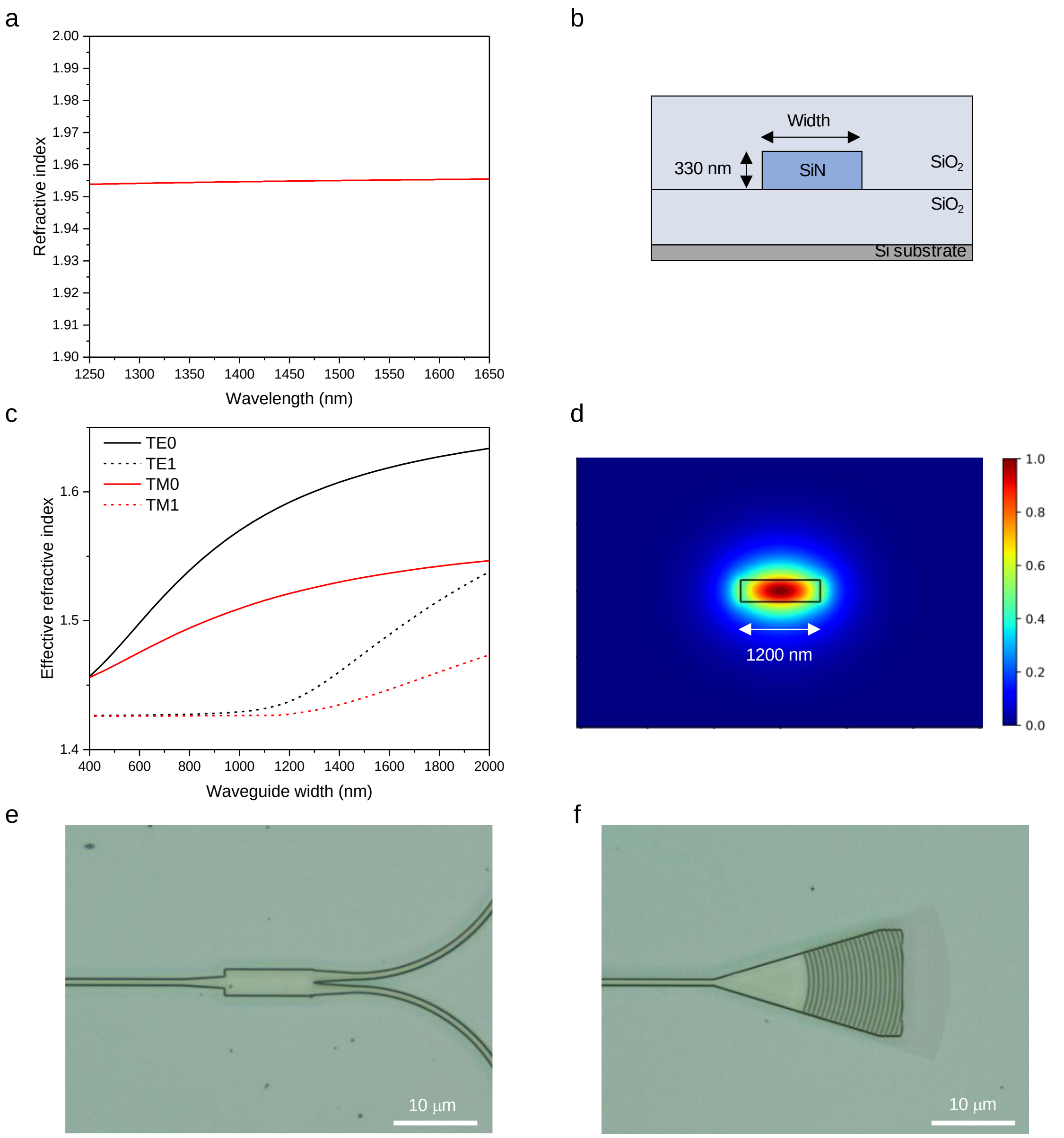

Fig. S2. **Analysis of SiN waveguide and plan-view images of SiN waveguide components. a,** Refractive index of the SiN layer measured by spectroscopic ellipsometry. **b,** Cross-sectional schematic of a SiN waveguide for numerical analysis. **c,** Effective refractive index of the fundamental and first TE and TM modes as functions of waveguide width. **d,** Mode profile of the fundamental TE mode when the waveguide width is 1200 nm. **e,** Plan-view microscopy image of the MMI coupler for AMZI. **f,** Plan-view microscopy image of the grating coupler.

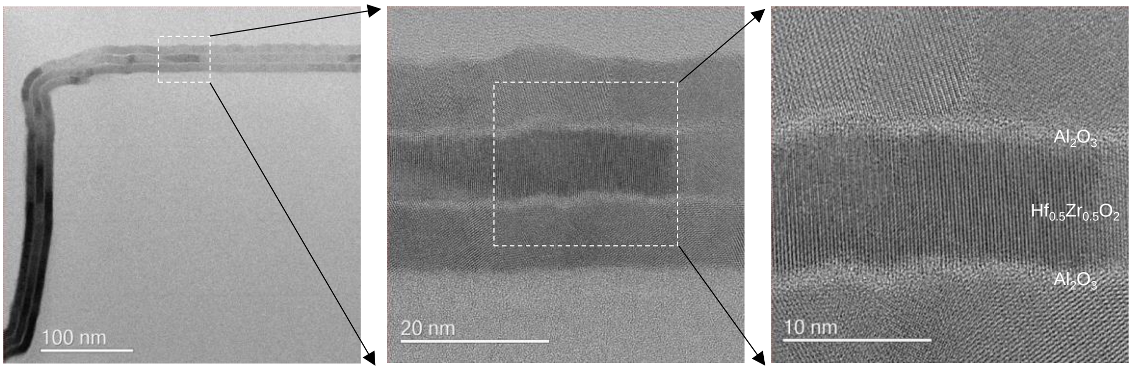

Fig. S3. **Cross-sectional TEM images of $Hf_{0.5}Zr_{0.5}O_2/Al_2O_3$ stacks deposited on the SiN waveguide.** Three polycrystalline $HfZrO_2$ layers separated by Al_2O_3 interlayers are clearly observed.

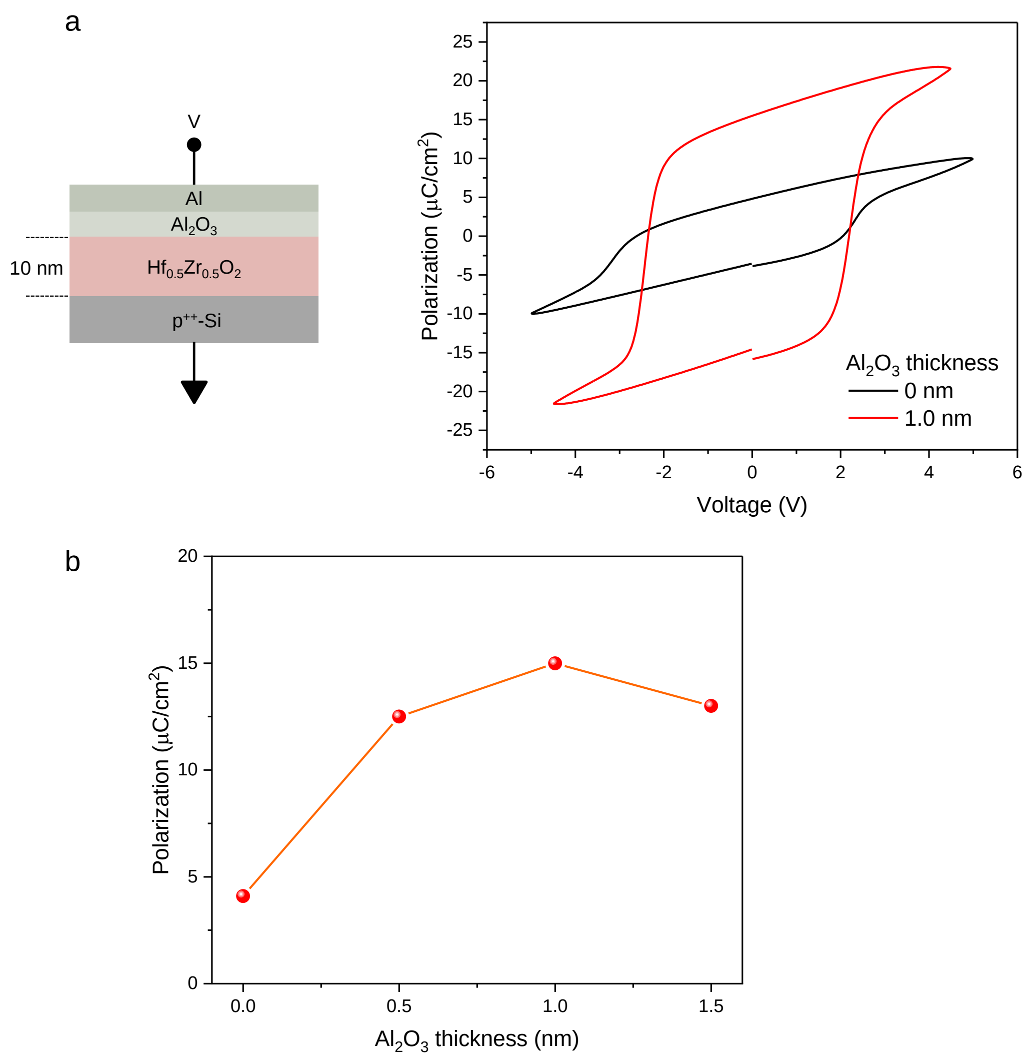

Fig. S4. **Ferroelectric properties of $Hf_{0.5}Zr_{0.5}O_2$ with Al_2O_3 capping layer. a,** PV characteristics of metal–insulator–semiconductor capacitors composed of a 10-nm-thick $Hf_{0.5}Zr_{0.5}O_2$ single layer fabricated on a heavily doped p-type Si substrate. **b,** Remanent polarization as a function of Al_2O_3 thickness. As the thickness of the Al_2O_3 capping layer increases, the remanent polarization increases and saturates for a 1-nm-thick Al_2O_3.

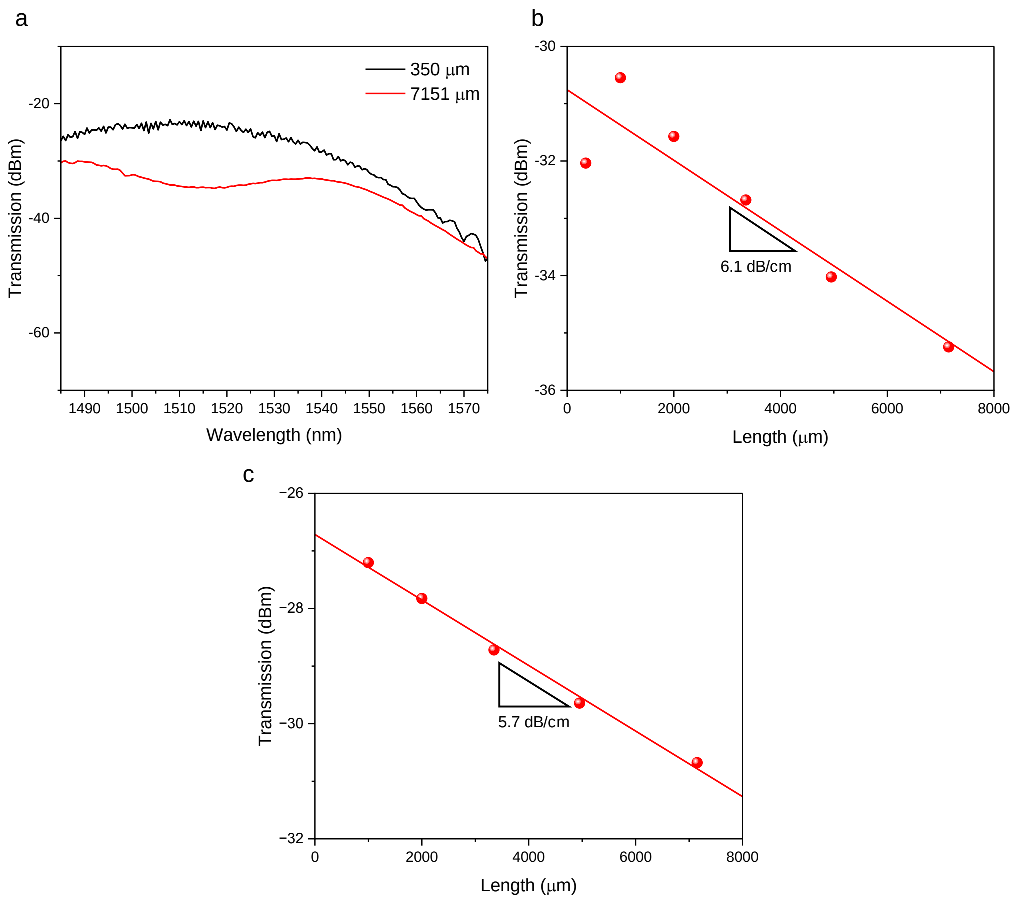

Fig. S5. **Transmission properties of straight SiN waveguides with grating couplers. a,** Transmission spectra of the SiN waveguides with lengths of 350 μm and 7151 μm. The observed attenuation in the wavelength range of 1500–1530 nm for the 7151-μm-long device is attributed to the absorption caused by the N–H bonds in SiN. **b,** Transmission of SiN waveguide with SiO_2 cladding at a wavelength of 1550 nm as a function of device length. The propagation loss is 6.1 dB/cm. **c,** Transmission of SiN waveguide with $Hf_{0.5}Zr_{0.5}O_2/Al_2O_3$ stacks at a wavelength of 1550 nm as a function of device length. The propagation loss is 5.7 dB/cm.

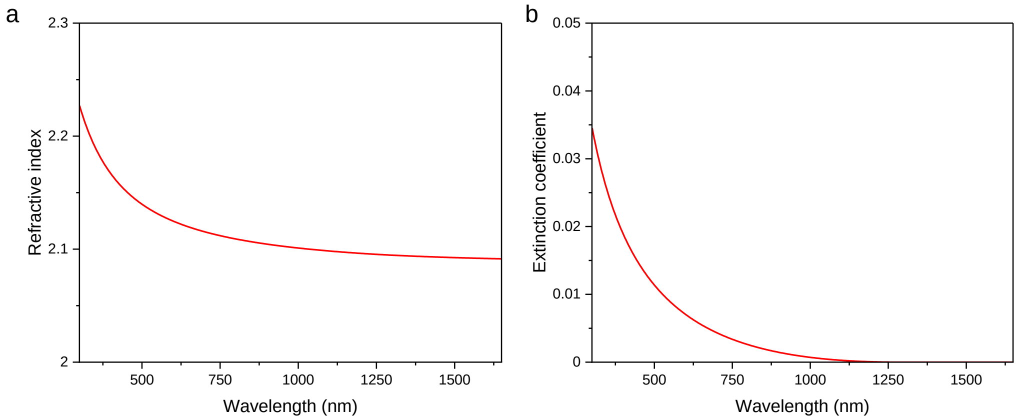

Fig. S6. **Ellipsometry measurements of** a 10-nm-thick $Hf_{0.5}Zr_{0.5}O_2$ film**. a,** Refractive index spectrum. **b,** Extinction coefficient spectrum.

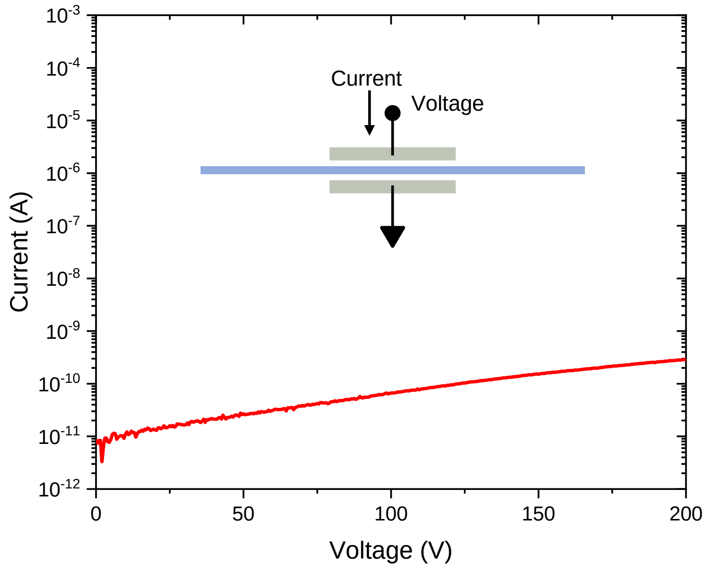

Fig. S7. **Current–voltage curve of 10-mm-long optical phase shifter.** The leakage current is less than 300 pA even at 200 V, suggesting that there is no significant TO effect during the measurement of the phase shift.

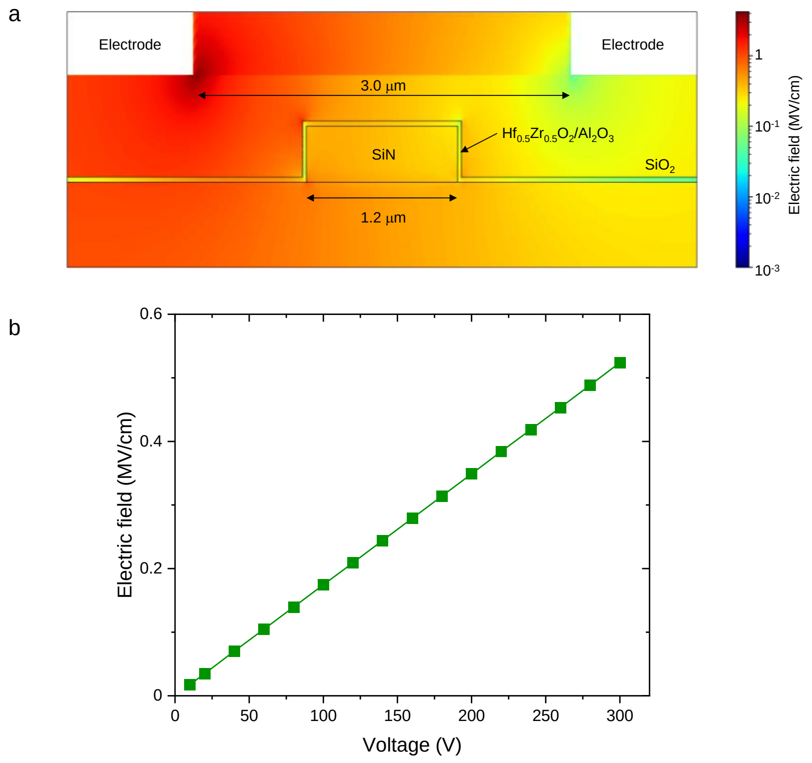

Fig. S8. **Simulation results of an external electric field. a,** Distribution of an external electric field when applying 200 V between two electrodes. **b,** Electric field atop the SiN waveguide. The external electric field is proportional to an applied voltage and reaches approximately 0.35 MV/cm at 200 V.

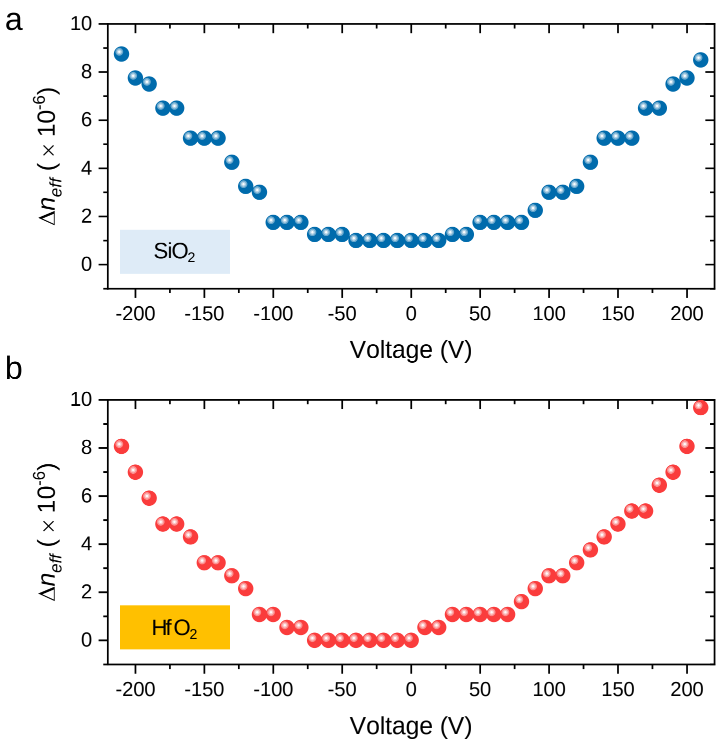

Fig. S9. **Change in the effective refractive index in SiO_2 and HfO_2 devices when voltage is swept between +210 and -210 V. a,** Change in the effective refractive index of the SiO_2 device. **b,** Change in the effective refractive index of the HfO_2 device. Both SiO_2 and HfO_2 exhibit the positive refractive index change that is proportional to the square of the applied voltage. The refractive index change can be attributed to the Kerr effect in SiN.

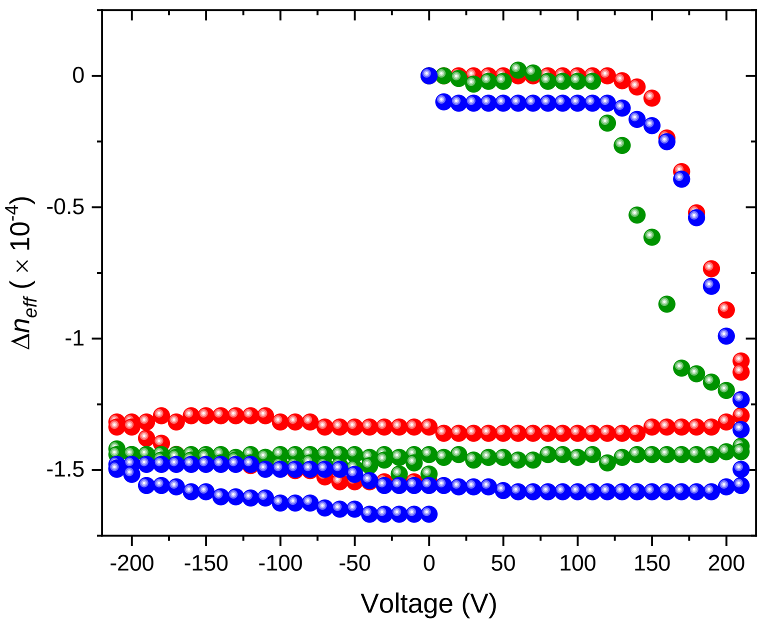

Fig. S10. **Variation in effective refractive index change when a voltage is swept between +210 and -210 V.** Three samples exhibit almost identical negative unidirectional refractive index change, indicating the reproducibility of nonvolatile optical phase shift.

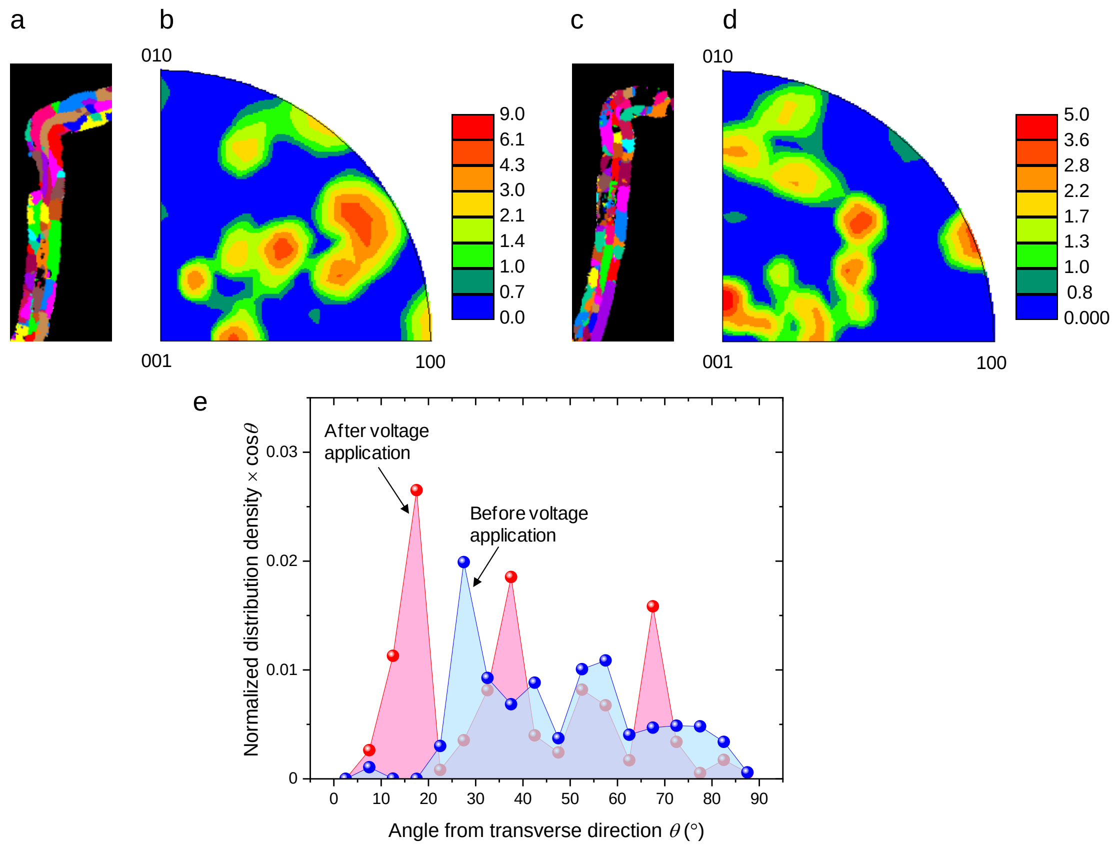

Fig. S11. **Automated crystal orientation mapping of the $Hf_{0.5}Zr_{0.5}O_2$ layers at the sidewall of the SiN waveguide. a,** Grain map and **b,** inverse pole figure of the orthorhombic $Hf_{0.5}Zr_{0.5}O_2$ layer before voltage application. **c,** Grain map and **d,** inverse pole figure of the orthorhombic $Hf_{0.5}Zr_{0.5}O_2$ layer after voltage application. **e,** Product of the normalized distribution density of the orientation angle θ of the orthorhombic phase and $\cos\theta$. The polarization axis rotates toward the direction of an external electric field owing to the voltage application, which contributes to the change in refractive index. The total area under the distribution curve, corresponding to the average of the transverse component, increased by approximately 20% after the voltage application.

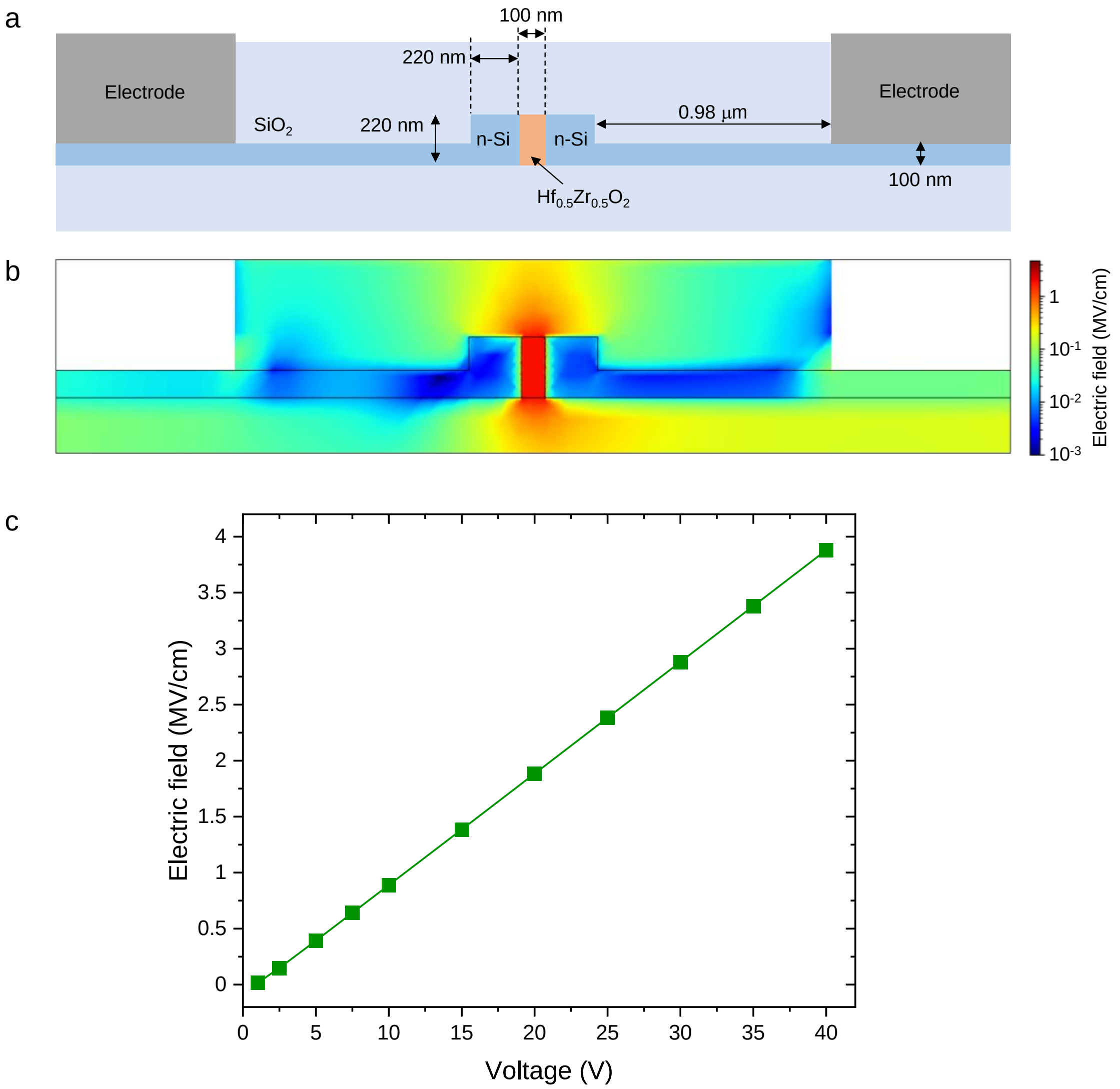

Fig. S12. **Simulation results of an external electric field in Si slot waveguide with HZO in the slot gap. a,** Cross-sectional schematic of slot waveguide-based device. The Si slot waveguide consists of n-doped Si layers. A bias voltage is applied to the slot gap through the n-doped Si layers. **b,** Distribution of an external electric field in the slot gap when applying 20 V between two electrodes. The external electric filed is concentrated in the slot gap. **c,** Electric field in the slot gap as a function of applied voltage. When an applied voltage is greater than 10 V, the external electric field exceed 1 MV/cm, which is comparable to the coercive electric field of HZO.

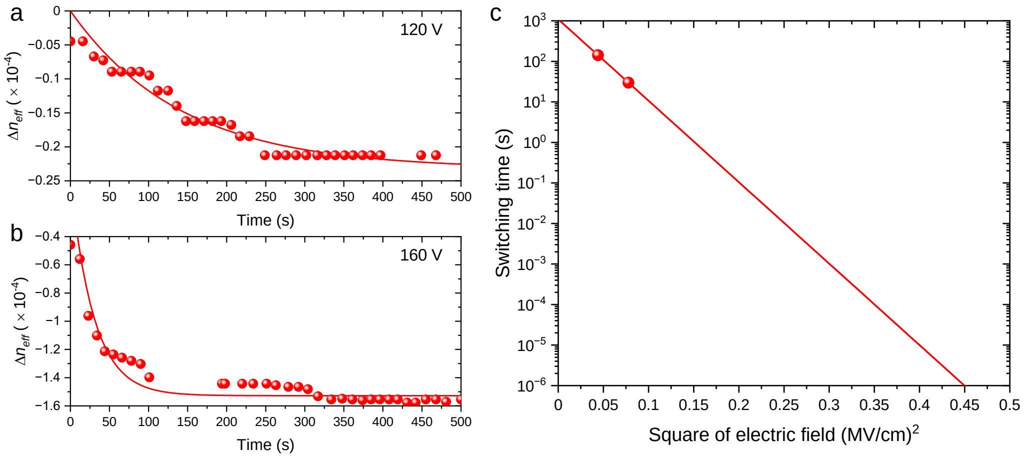

Fig. S13. **Transient measurement results of the SiN device. a,** Transient of refractive index change when applying 120 V. An exponential decay fitting curve is indicated by the solid line. **b,** Transient of refractive index change when applying 160 V. **c,** Switching time as a function of the square of the external electric field. According to the numerical fitting curve based on the nucleation-dominated switching model, the anticipated switching time falls below 1 μs when the external electric field exceeds approximately 0.7 MV/cm.
